# Lung eicosanoids in perinatal rats with congenital diaphragmatic hernia

**DOI:** 10.1080/09629359791910

**Published:** 1997-02

**Authors:** H. Ijsselstijn, F. J. Zijlstra, J. P. M. Van Dijk, J. C. De Jongste, D. Tibboel

**Affiliations:** 1 Department of Paediatric Surgery Division of Respiratory Medicine Erasmus University Rotterdam and University Hospital/Sophia Children's Hospital Dr. Molewaterplein 60 Rotterdam 3015 GJ The Netherlands; 2 Department of Paediatrics Division of Respiratory Medicine Erasmus University Rotterdam and University Hospital/Sophia Children's Hospital Dr. Molewaterplein 60 Rotterdam 3015 GJ The Netherlands; 3 Department of Pharmacology Erasmus University Rotterdam and University Hospital/Sophia Children's Hospital Dr. Molewaterplein 60 Rotterdam 3015 GJ The Netherlands

## Abstract

Abnormal levels of pulmonary eicosanoids have been reported in infants with persistent pulmonary hypertension (PPH) and congenital diaphragmatic hernia (CDH). We hypothesized that a dysbalance of vasoconstrictive and vasodilatory eicosanoids is involved in PPH in CDH patients. The levels of several eicosanoids in lung homogenates and in bronchoalveolar lavage fluid of controls and rats with CDH were measured after caesarean section or spontaneous birth. In controls the concentration of the stable metabolite of prostacyclin (6-keto-PGF_1α_), thromboxane A_2_ (TxB_2_), prostaglandin E_2_ (PGE_2_), and leukotriene B_4_ (LTB_4_) decreased after spontaneous birth. CDH pups showed respiratory insufficiency directly after birth. Their lungs had higher levels of 6- keto-PGF_1α_, reflecting the pulmonary vasodilator prostacyclin (PGI_2_), than those of controls. We conclude that in CDH abnormal lung eicosanoid levels are present perinatally. The elevated levels of 6-keto-PGF_1α_ in CDH may reflect a compensation mechanism for increased vascular resistance.


Mediators of Inflammation, **6**, 39–45 (1997)

### Lung eicosanoids in perinatal rats with congenital diaphragmatic hernia

H. IJsselstijn,[1,2] F. J. Zijlstra,[3] J. P. M. van Dijk,[3] J. C. de Jongste[2] and D. Tibboel[1,CA]

Departments of [1]Paediatric Surgery, [2]Paediatrics, Division of Respiratory Medicine, [3]Pharmacology, Erasmus University Rotterdam and University Hospital/Sophia Children's Hospital, Dr. Molewaterplein 60, 3015 GJ Rotterdam, The Netherlands

[CA]Corresponding Author
Tel: (+31) 10 4636567
Fax: (+31) 10 4636288

**Abnormal levels of pulmonary eicosanoids have been reported in infants with persistent pulmonary hypertension (PPH) and congenital diaphragmatic hernia (CDH). We hypothesized that a dysbalance of vasoconstrictive and vasodilatory eicosanoids is involved in PPH in CDH patients. The levels of several eicosanoids in lung homogenates and in bronchoalveolar lavage fluid of controls and rats with CDH were measured after caesarean section or spontaneous birth. In controls the concentration of the stable metabolite of prostacyclin (6-keto-PGF$_{1\alpha}$), thromboxane $A_2$ ($TxB_2$), prostaglandin $E_2$ ($PGE_2$), and leukotriene $B_4$ ($LTB_4$) decreased after spontaneous birth. CDH pups showed respiratory insufficiency directly after birth. Their lungs had higher levels of 6-keto-PGF$_{1\alpha}$, reflecting the pulmonary vasodilator prostacyclin ($PGI_2$), than those of controls. We conclude that in CDH abnormal lung eicosanoid levels are present perinatally. The elevated levels of 6-keto-PGF$_{1\alpha}$ in CDH may reflect a compensation mechanism for increased vascular resistance.**

**Key words:** Diaphragmatic hernia, Leukotrienes, Lung, Newborn animals, Prostaglandins, Pulmonary hypertension, Thromboxanes

### Introduction

Eicosanoids are arachidonic acid metabolites which are produced in different tissues in human and animal species.[1] They have been studied extensively in relation to the perinatal pulmonary circulation, and have been implicated in several physiologic and pathologic conditions such as persistent pulmonary hypertension (PPH).[2–7]

Prostacyclin ($PGI_2$), prostaglandin $E_2$ ($PGE_2$), and thromboxane $A_2$ ($TxA_2$) are all generated via the cyclooxygenase pathway; the latter has a pulmonary vasoconstricting activity, whereas the other two are pulmonary vasodilators.[6] Increased circulating levels of $PGE_2$ may contribute to the pathogenesis of patent ductus arteriosus.[8] Leukotrienes, which are formed by the 5-lipoxygenase pathway, may have a key function in maintaining the elevated pulmonary vascular resistance in the fetus,[6,9] although this could not be confirmed in other studies.[7,10]

Children with congenital diaphragmatic hernia (CDH) have abnormal morphological development of lungs and intrapulmonary blood vessels.[11,12] The high neonatal mortality and morbidity is ascribed to the extent of lung hypoplasia and PPH.[13] Increased levels of leukotrienes, and of metabolites of $PGI_2$ and $TxA_2$ have been reported in plasma and in bronchoalveolar lavage (BAL) fluid of both PPH patients without CDH and children with CDH.[14–21]

We hypothesized that the pulmonary vascular abnormalities in CDH cause abnormal transition of the pulmonary circulation at birth, associated with a dysbalance of vasoconstrictive and vasodilatory eicosanoids. Therefore, we studied the content of different eicosanoids—metabolites from the cyclooxygenase and one from the lipoxygenase pathway—in lung homogenates and in BAL fluid of perinatal rats with CDH.[22] The pulmonary vascular abnormalities in these rat pups strongly resemble those of children with CDH.[23]

### Materials and Methods

### Animal model

Female Sprague-Dawley rats (Harlan Olac, UK) were mated during 1 h (day 0 of gestation). Nine of 18 pregnant rats received 100 mg of 2,4-dichloro-phenyl-p-nitrophenylether (Nitrofen: Rohm Haas Company, Philadelphia, PA) in 1 ml of olive oil orogastrically under light ether anaesthesia on day 10 of gestation;[22] the remaining nine rats provided control pups. Nitrofen induces a large left-sided diaphragmatic defect

© 1997 Rapid Science Publishers with severe lung hypoplasia in up to 80% of the offspring using this regimen.[22] Food and water were supplied *ad libitum* during the whole period of pregnancy. Nine pregnant dams were anaesthetized by inhalation of diethylether and a caesarean section was performed on day 22 (nitrofen-exposed litters $n = 5$; control litters $n = 4$). While they were kept in the membranes to prevent any breathing, the fetuses died after cervical intersection with a needle, and were weighed. Only rat pups that could be processed within the first 30 min of anaesthesia were included. In the remaining litters (nitrofen-exposed $n = 4$, and controls $n = 5$) spontaneous birth on day 22–23 was awaited; within 5–10 min after birth they were killed as described above, and weighed. The presence of a diaphragmatic defect in all nitrofen-exposed rat pups was revealed by autopsy. To obtain a homogeneous group only nitrofen-exposed rat pups with left-sided or bilateral diaphragmatic defects with concomitant severe lung hypoplasia were included, and nitrofen-exposed pups with small right-sided defects or without CDH were excluded. Thus four different groups were studied: CDH rat pups after caesarean section or born spontaneously, and control pups after caesarean section or born spontaneously. Either BAL procedure or dissection of the lungs for preparation of homogenates was then performed.

### Lung homogenates

The lungs were removed, stripped of non-pulmonary tissue, separated, weighed, frozen in liquid $N_2$, and stored at −70°C until further processed. They they were homogenized in 1 ml in Krebs-solution, and centrifuged at 2500 ***g***. The content of eicosanoids and protein was measured in the supernatant. Ten samples were obtained in CDH pups after caesarean section and four in spontaneously born pups. In controls the numbers were $n = 11$ and $n = 23$, respectively.

### BAL procedure

After opening of the abdominal cavity and assessment of the diaphragmatic defect in the nitrofen-exposed pups, the thorax was opened, and a tracheotomy was performed. A polyethylene catheter (Portex, UK; outer diameter 0.61 mm or 1.0 mm, inner diameter 0.28 or 0.5 mm, for CDH pups and controls respectively) was inserted into the trachea and ligated. A 1 ml-syringe with NaCl 0.9%, heated to 37°C, was connected to the catheter, and the lungs were washed as previously described.[24] In CDH pups the lungs were washed with seven to 10 times 0.05 to 0.1 ml. Lungs from control pups were washed four times with 0.25 to 0.45 ml, until 1 ml of fluid had been recovered. Samples that were visibly contaminated with blood were excluded. Ten samples were obtained in each CDH group, and 13 samples in each control group. The BAL fluid was directly frozen in liquid $N_2$ and stored at −70°C until assay.

### Measurement of eicosanoids and total protein

The following eicosanoids were measured by radioimmunoassay: 6-keto-$PGF_{1\alpha}$ (the stable metabolite of prostacyclin), $PGE_2$, $TxB_2$ (the stable metabolite of $TxA_2$), all three generated by the cyclooxygenase pathway, and leukotriene $B_4$ ($LTB_4$), a lipoxygenase-derived metabolite of arachidonic acid. All assays were performed as described in detail previously.[25] Total protein was measured by ELISA at 595 nm using a commercially available protein reagent and protein standard (Instruchemie B.V., Hilversum, the Netherlands).

### Data analysis

All eicosanoid levels are expressed as pg/μg protein (mean ± SEM), unless stated otherwise. Differences between groups were tested by Student's *t*-test or by the non-parametric Mann–Whitney test if appropriate. Statistical significance was assumed at 5% level.

### Results

All spontaneously born control pups had a regular respiration rate and were pink within minutes after birth. Respiratory insufficiency with gasping and cyanosis was observed in rat pups with CDH, but not in controls, directly after birth.

The lung weights in spontaneously born control pups were significantly lower than those in controls delivered by caesarean section (Table 1; $P < 0.001$). This was not the case in the CDH pups: the lung weights were similar in both groups (Table 1). Control lungs were significantly heavier than lungs in CDH ($P < 0.001$).

### Results in lung homogenates

First, data from the left and the right lungs in all groups were analysed separately to determine whether there were consistent differences in

**Table 1**. Lung weights and total amount of eicosanoids in lung homogenates of controls and CDH pups after delivery by caesarean section or after spontaneous birth

| | | Caesarean section | Spontaneous birth |
|---|---|---|---|
| Lung weight (mg) | Control | 149 ± 2 | 115 ± 4[b] |
| | CDH | 62 ± 2[a] | 62 ± 3[a] |
| 6-keto-$PGF_{1\alpha}$ (pg) | Control | 4340 ± 210 | 3260 ± 160[b] |
| | CDH | 7830 ± 320[a] | 7670 ± 270[a] |
| $TxB_2$ (pg) | Control | 27620 ± 1600 | 21560 ± 850[b] |
| | CDH | 21070 ± 1320[a] | 19970 ± 880 |
| $PGE_2$ (pg) | Control | 42110 ± 2210 | 30410 ± 1450[b] |
| | CDH | 27750 ± 2050[a] | 24690 ± 1630[a] |
| $LTB_4$ (pg) | Control | 3410 ± 180 | 2950 ± 130[b] |
| | CDH | 2160 ± 150[a] | 2610 ± 350 |

All data are expressed as mean ± SEM. The numbers per group are: n = 11 and n = 23 for the controls delivered by caesarean section and by spontaneous birth, respectively; n = 10 and n = 4 for the respective CDH groups.
[a]Significantly different from control, same delivery mode; P < 0.05.
[b]Significantly different from caesarean section, same group; P < 0.05.

eicosanoid levels between the ipsilateral and contralateral lungs in CDH (data not shown). This was not the case, however, and data from both lungs were therefore pooled.

In CDH pups protein per mg wet lung weight was higher than in controls: 28.8 ± 0.8 μg and 27.5 ± 1.1 μg after caesarean section and spontaneous delivery in CDH, respectively, and 13.5 ± 0.1 μg and 18.5 ± 0.6 μg in controls, respectively ($P < 0.001$). In controls the protein content per mg lung weight was significantly lower in pups who were delivered by caesarean section than in spontaneously born pups ($P < 0.001$), but this was not true for the total protein content in both lungs (2020 ± 9 μg after caesarean section and 2060 ± 24 μg after spontaneous birth). In all control pups the total amount of protein was higher than in CDH pups, whose lungs contained 1760 ± 12 μg and 1710 ± 8 μg protein in the respective groups ($P < 0.001$).

The eicosanoid concentrations per μg protein measured in the lung homogenates are shown in Fig. 1. In controls the concentrations of all eicosanoids per μg protein (Fig. 1A–D) and the total amount of eicosanoids (Table 1) were significantly lower in spontaneously born pups, compared with those in the caesarean section group. In CDH pups the eicosanoid levels were not affected by the delivery mode; this was also the case for the eicosanoid content per mg lung weight (data not shown).

The levels of 6-keto-$PGF_{1\alpha}$ per μg protein (Fig. 1A; $P < 0.001$) and the total amount of 6-keto-$PGF_{1\alpha}$ (Table 1; $P < 0.001$) were significantly higher in CDH than in controls. In addition, the ratio of 6-keto-$PGF_{1\alpha}$ to $TxB_2$ was calculated for each group; in the caesarean section group it was 0.38 ± 0.03 and 0.16 ± 0.01 for CDH and controls, respectively ($P < 0.001$), and for spontaneously born rat pups 0.39 ± 0.02 and 0.16 ± 0.01, respectively ($P < 0.001$).

Controls born by caesarean section had higher total $TxB_2$ than CDH pups (Table 1; $P = 0.006$) and a tendency towards higher $TxB_2$ per μg protein (Fig. 1B; $P = 0.08$). No such differences for $TxB_2$ were observed in the spontaneously born rat pups. $PGE_2$ per μg protein was significantly higher in control pups delivered by caesarean section than in CDH pups (Fig. 1C; $P = 0.003$). The total amounts of $PGE_2$ were higher in controls than in CDH pups, irrespective of the delivery mode (Table 1). The concentration of $LTB_4$ per μg protein (Fig. 1D) and the total amount of $LTB_4$ (Table 1) were significantly higher in controls than in CDH pups after caesarean section ($P < 0.001$), whereas both groups showed similar $LTB_4$ levels after spontaneous delivery.

### Eicosanoids in BAL fluid

In BAL fluid a wide range of eicosanoid concentrations was observed. In controls the concentrations per ml BAL fluid of 6-keto-$PGF_{1\alpha}$, $TxB_2$, and $LTB_4$ were higher after spontaneous birth than after caesarean section (Table 2; $P = 0.01$, 0.06, and < 0.001, respectively). However, after correction for dilution, with total protein as marker, only $LTB_4$ was significantly higher after spontaneous birth (Table 2; $P = 0.02$). CDH pups showed higher uncorrected concentration levels of $TxB_2$ and $LTB_4$ in spontaneously born rats compared with pups delivered by caesarean section (Table 2; $P = 0.04$ and 0.05, respectively). The same volumes in CDH pups were so small that the protein concentration could only be measured in eight samples ($n = 4$ per group).

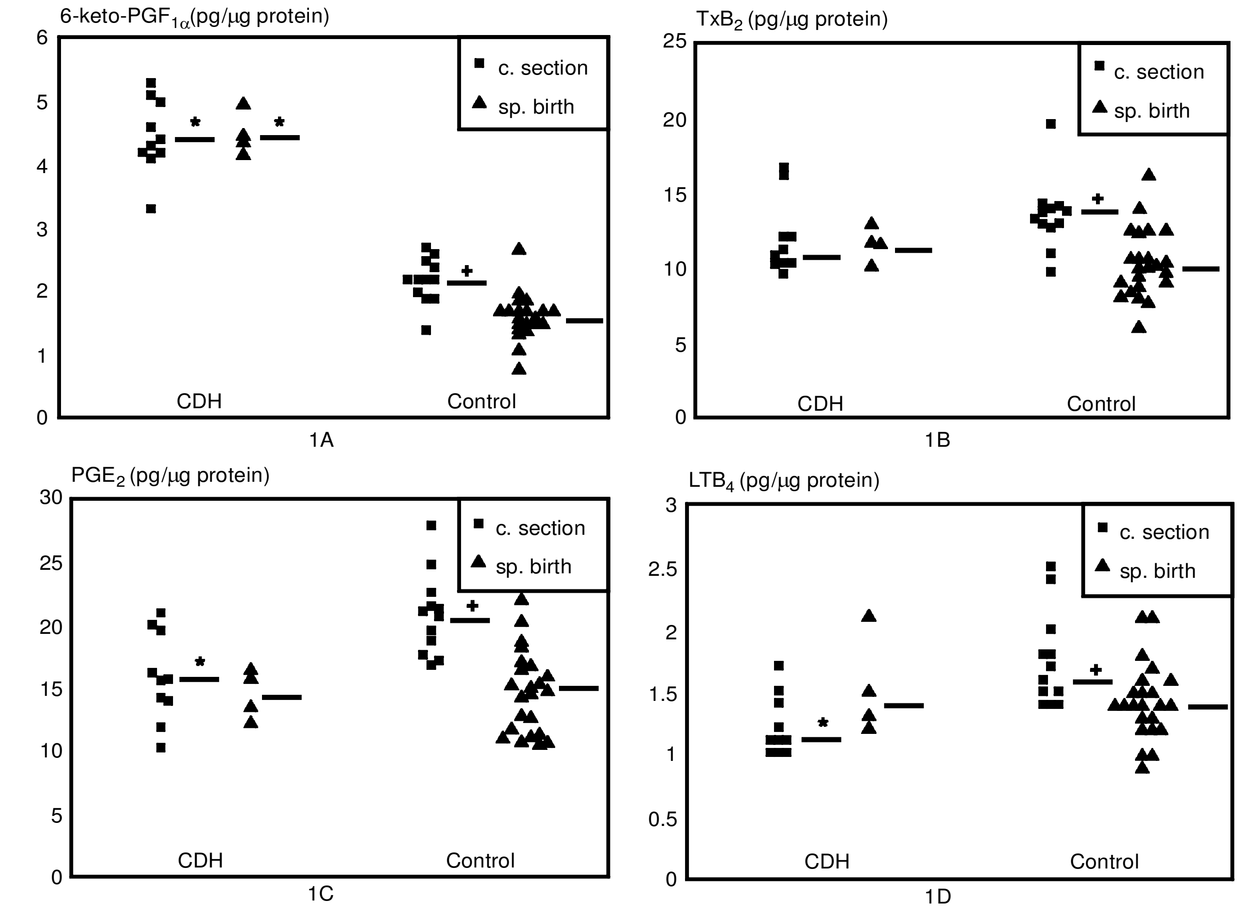

FIG. 1. Concentration of 6-keto-$PGF_{1\alpha}$ (1A), $TxB_2$ (1B), $PGE_2$ (1C), and $LTB_4$ (1D) per μg protein in lung homogenates of rat pups with CDH and controls delivered by caesarean section (squares) or spontaneously born pups (triangles). Median is indicated for each group. *Significantly different from controls; similar mode of delivery; $P < 0.01$. $^{+}$Significantly different from controls after spontaneous birth; $P < 0.02$.

**Table 2**. Eicosanoids in BAL fluid of controls and CDH pups after delivery by caesarean section or after spontaneous birth

| | | Caesarean section | Spontaneous birth |
|---|---|---|---|
| 6-keto-$PGF_{1\alpha}$ (pg/ml) | Control | 171 (64–303) | 278 (102–743)[a] |
| | CDH | 207 (110–521) | 243 (172–617) |
| 6-keto-$PGF_{1\alpha}$ (pg/μg protein) | Control | 1.72 (0.58–14.8) | 1.43 (1.06–4.04) |
| | CDH | 5.53 (1.88–38.9) | 1.81 (1.12–3.52) |
| $TxB_2$ (pg/ml) | Control | 100 (23–372) | 182 (78–496) |
| | CDH | 65 (6–140) | 96 (19–305)[a] |
| $TxB_2$ (pg/μg protein) | Control | 1.19 (0.47–5.33) | 1.18 (0.3–2.75) |
| | CDH | 1.62 (0.16–6.72) | 0.69 (0.43–2.5) |
| $LTB_4$ (pg/ml) | Control | 15 (5–105) | 162 (25–661)[a] |
| | CDH | 42 (14–194) | 100 (51–200)[a,b] |
| $LTB_4$ (pg/μg protein) | Control | 0.24 (0.08–6.9) | 0.92 (0.12–2.57)[a] |
| | CDH | 0.23 (0.19–2.0) | 0.87 (0.38–0.99) |

All values are expressed as median (range). Data shown per ml BAL fluid are n = 10 for each CDH group and n = 13 per control group. Data shown per μg protein are n = 4 for each CDH group, n = 13 for controls delivered by caesarean section, and n = 10 for spontaneously born controls.

[a]Significantly different from caesarean section in the same group; $P < 0.05$.

[b]Significantly different from spontaneously born CDH pups; $P < 0.05$.

The ratio of 6-keto-$PGF_{1\alpha}$ and $TxB_2$ in BAL fluid was significantly higher in CDH pups than in controls who were delivered by caesarean section ($7.93 \pm 2.95$ and $2.22 \pm 0.5$, respectively; $P = 0.02$). A similar tendency was observed for the spontaneously born rat pups ($3.63 \pm 1.13$ for CDH, and $1.48 \pm 0.32$ for controls; $P = 0.06$).

### Discussion

In the present study higher levels of 6-keto-$PGF_{1\alpha}$, the stable metabolite of the pulmonary vasodilator $PGI_2$, were found in the lungs of CDH pups than in those of controls, irrespective of the mode of delivery. Lungs of CDH pups had similar or lower levels of $TxB_2$, $PGE_2$, and $LTB_4$ than control pups. All eicosanoids studied were higher in the lungs of control pups delivered by caesarean section than in those born spontaneously; this was not the case in CDH pups.

The lower lung weights in spontaneously born controls compared with those delivered by caesarean section probably indicate that lung fluid was absorbed to a large extent during the first adequate breaths. The gasping, irregular breathing movement in the spontaneously born CDH pups have been insufficient to overcome the pressure that is needed to initiate lung expansion and to provide adequate lung aeration and absorption of lung fluid,[26,27] thus explaining the similar lung weights in both CDH groups.

We studied the eicosanoid concentration both in lung homogenates and in BAL fluid to determine whether the concentration in BAL fluid adequately reflects the situation in the lung tissue. We found widely varying eicosanoid concentrations in BAL fluid of the neonatal rat pups. After correction for protein, only the concentration of 6-keto-$PGF_{1\alpha}$ in control pups showed comparable results between BAL fluid and lung homogenates. The concentration of $TxB_2$ was generally 10 times higher in lung homogenates than in BAL fluid, which suggests that thromboxane is mainly present in the pulmonary vasculature and not into the airspaces. The same may be true for the concentration of $LTB_4$ during intrauterine life. Our data support earlier observations that the eicosanoid content in the pulmonary vasculature is more adequately reflected in tissue homogenates than in BAL fluid.[7] However, the ratio of 6-keto-$PGF_{1\alpha}$ and $TxB_2$ was significantly higher in lung homogenates and in BAL fluid of CDH pups than in that of controls, suggesting that this parameter in BAL fluid reflects the values in lung tissue. A high ratio of 6-keto-$PGF_{1\alpha}$ and $TxB_2$ was also found in BAL fluid of two CDH patients with evidence of PPH (unpublished data).

During normal transition from intrauterine to extrauterine life, the pulmonary vascular resistance rapidly declines within the first 30 s, and declines more slowly over the next 10–20 min.[2] The first phase occurs irrespective of prostaglandin synthase blockade by indomethacin,[2] but several studies in lambs and goats indicate that a transient prostacyclin production in the lungs, which is stimulated by tissue stress during establishment of gaseous ventilation and rhythmic ventilation,[4] is important to sustain further pulmonary vasodilatation within the first hours after birth.[2–4,7]

The lower levels of all eicosanoids in lung tissue of normal controls compared with CDH pups following spontaneous birth in this study seem to contradict the earlier findings in newborn lambs and goats.[2–4,7] Perhaps the described loss of prostacyclin from the lungs shortly before birth[3] continued immediately after birth, and the rat pups died before the prostacyclin concentration began to increase. However, the CDH pups could not survive much longer without artificial ventilation and supplemental oxygen.

Persistent pulmonary hypertension is a serious problem in neonatology which largely contributes to the neonatal mortality and morbidity in isolated cases of PPH,[28] and in children with CDH.[13] Improvement of oxygenation parameters in some children with PPH has been reported after intravenous or inhaled administration of prostacyclin,[29,30] although other patients seem irresponsive to vasodilator therapy like prostacyclin[29] or inhaled nitric oxide.[28]

Increased levels of 6-keto-$PGF_{1\alpha}$, $TxB_2$, $PGE_2$, and leukotrienes have been reported in plasma and in bronchoalveolar lavage fluid of PPH patients[14,17,19,21] and in plasma of CDH patients with PPH.[15,16,21] A decrease in all eicosanoid levels was observed during clinical improvement, especially in patients who were being treated with extracorporeal membrane oxygenation.[16,21] It has been suggested that $LTC_4$, $LTD_4$ and $TxB_2$ have a pulmonary vasoconstricting activity, whereas 6-keto-$PGF_{1\alpha}$ opposes the hypoxic vasoconstriction.[31]

In a fetal lamb model of chronic intrauterine pulmonary hypertension[32] increased pulmonary levels of 6-keto-$PGF_{1\alpha}$ and $TxB_2$ were detected shortly before, and 2 h after birth.[7] Our study did not reveal significant differences in eicosanoid content in the lungs of the CDH rats during transition from intrauterine to extrauterine life; this may be due to the short period of survival after birth and the lack of adequate respiratory movements.

We found increased levels of 6-keto-$PGF_{1\alpha}$ per μg protein, and decreased levels of $PGE_2$ and $LTB_4$ in lung tissue of CDH pups in the caesarean section group. Surprisingly, the concentration of $TxB_2$ per μg protein was similar in

CDH pups and in controls. We assumed that a certain total amount of eicosanoids is important to exert a local effect, and we therefore determined the total eicosanoid content in both lungs of CDH and control pups. The results were similar to the data that were corrected for protein. The increased ratio of 6-keto-$PGF_{1\alpha}$ and $TxB_2$ in CDH pups compared with control pups confirms the presence of a dysbalance in vasoactive mediators, which is in favour of the pulmonary vasodilator. It has been suggested that prostaglandin generation in the pulmonary vasculature may reduce the pulmonary vascular pressure response to hypoxia.[31] Cott and coworkers[33] showed in adult rats that alveolar type II cells are capable of producing high levels of 6-keto-$PGF_{1\alpha}$ and $PGE_2$ *in vitro*, whereas $TxB_2$ and $LTB_4$ are mainly produced by alveolar macrophages. Brandsma and coworkers[34] reported more type II cells that showed retarded differentiation in CDH lungs. The relatively higher number of type II cells may be responsible for the increased 6-keto-$PGF_{1\alpha}$ content in the CDH lungs.

In conclusion, the present study shows different eicosanoid profiles in lungs of perinatal rats with and without CDH. The most striking findings are the elevated concentration of 6-keto-$PGF_{1\alpha}$, and the increased ratio of 6-keto-$PGF_{1\alpha}$ and $TxB_2$ in CDH lungs. This is the first study of lung eicosanoids in perinatal animals with abnormal lung development. From the present data it is not clear whether the balance which is in favour of 6-keto-$PGF_{1\alpha}$, the stable metabolite of the pulmonary vasodilator $PGI_2$, compensates for an increased pulmonary vascular tone or that it reflects delayed cell differentiation in CDH. Our findings give reason to assume that lungs of CDH patients with PPH already contain increased levels of prostacyclin at birth and that the administration of exogenous vasodilators will not be helpful to decrease the pulmonary vascular resistance in those patients.

### References

1. Moncada S, Vane JR. Pharmacology and endogenous roles of prostaglandin endoperoxides, thromboxane $A_2$, and prostacyclin. *Pharmacol Rev* 1979; **30**: 293–331.
2. Leffler CW, Tyler TL, Cassin S. Effect of indomethacin on pulmonary vascular response to ventilation of fetal goats. *Am J Physiol* 1978; **234**: H346–H351.
3. Leffler CW, Hessler JR, Green RS. The onset of breathing at birth stimulates pulmonary vascular prostacyclin synthesis. *Pediatr Res* 1984; **18**: 938–942.
4. Leffler CW, Hessler JR, Green RS. Mechanism of stimulation of pulmonary prostacyclin synthesis at birth. *Prostaglandins* 1984; **28**: 877–887.
5. Mitchell MD. Prostaglandins during pregnancy and the perinatal period. *J Reprod Fert* 1981; **62**: 305–315.
6. Coceani F, Olley PM. Eicosanoids in the fetal and transitional pulmonary circulation. *Chest* 1988; **93**: 112S–117S.
7. Abman SH, Stenmark KR. Changes in lung eicosanoid content during normal and abnormal transition in perinatal lambs. *Am J Physiol* 1992; **262**: L214–L222.
8. Clyman RI, Mauray F, Roman C, Rudolph AM, Heymann MA. Circulating prostaglandin $E_2$ concentrations and patent ductus arteriosus in fetal and neonatal lambs. *J Pediatr* 1980; **97**: 455–461.
9. Cassin S. Role of prostaglandins, thromboxanes, and leukotrienes in the control of the pulmonary circulation in the fetus and newborn. *Sem Perinatol* 1987; **11**: 53–63.
10. Cassin S. The role of eicosanoids and endothelium-dependent factors in the regulation of the fetal pulmonary circulation. *J Lipid Med* 1993; **6**: 477–485.
11. Levin DL. Morphologic analysis of the pulmonary vascular bed in congenital left-sided diaphragmatic hernia. *J Pediatr* 1978; **107**: 457–464.
12. George DK, Cooney TP, Chiu BK, Thurlbeck WM. Hypoplasia and immaturity of the terminal lung unit (acinus) in congenital diaphragmatic hernia. *Am Rev Respir Dis* 1987; **136**: 947–950.
13. Molenaar JC, Bos AP, Hazebroek FWJ, Tibboel D. Congenital diaphragmatic hernia, what defect? *J Pediatr Surg* 1991; **26**: 248–254.
14. Stenmark KR, James SL, Voelkel NF, Toews WH, Reeves JT, Murphy RC. Leukotriene $C_4$ and $D_4$ in neonates with hypoxemia and pulmonary hypertension. *N Engl J Med* 1983; **309**: 77–80.
15. Ford WDA, James MJ, Walsh JA. Congenital diaphragmatic hernia: association between congenital pulmonary vascular resistance and plasma thromboxane concentrations. *Arch Dis Child* 1984; **59**: 143–146.
16. Stolar CJH, Dillon PW, Stalcup SA. Extracorporeal membrane oxygenation and congenital diaphragmatic hernia: modification of the pulmonary vasoactive profile. *J Pediatr Surg* 1985; **20**: 681–683.
17. Hammerman C, Lass N, Strates E, Komar K, Bui KC. Prostanoids in neonates with persistent pulmonary hypertension. *J Pediatr* 1987; **110**: 470–472.
18. Bos AP, Tibboel D, Hazebroek FWJ, Stijnen T, Molenaar JC. Congenital diaphragmatic hernia: impact of prostanoids in the perioperative period. *Arch Dis Child* 1990; **65**: 994–995.
19. Bui KC, Hammerman C, Hirschl RB, Snedecor SM, Cheng KJ, Chan L, Short BL, Bartlett RH. Plasma prostanoids in neonates with pulmonary hypertension treated with conventional therapy and with extracorporeal membrane oxygenation. *J Thorac Cardiovasc Surg* 1991; **101**: 973–983.
20. Nakayama DK, Motoyama EK, Evans R, Hannakan C. Relation between arterial hypoxemia and plasma eicosanoids in neonates with congenital diaphragmatic hernia. *J Surg Res* 1992; **53**: 615–620.
21. Dobyns EL, Westcott JY, Kennaugh JM, Ross MN, Stenmark KR. Eicosanoids decrease with successful extracorporeal membrane oxygenation therapy in neonatal pulmonary hypertension. *Am J Respir Crit Care Med* 1994; **149**: 873–880.
22. Kluth D, Kangah R, Reich P, Tenbrinck R, Tibboel D, Lambrecht W. Nitrofen-induced diaphragmatic hernias in rats: an animal model. *J Pediatr Surg* 1990; **25**: 850–854.
23. Tenbrinck R, Gaillard JLJ, Tibboel D, Kluth D, Lachmann B, Molenaar JC. Pulmonary vascular abnormalities in experimentally induced congenital diaphragmatic hernia in rats. *J Pediatr Surg* 1990; **27**: 862–865.
24. Brandsma AE, Tibboel D, Vulto IM, Egberts J, Ten Have-Opbroek AAW. Ultrastructural features of alveolar epithelial cells in the late fetal pulmonary acinus: a comparison between normal and hypoplastic lungs using a rat model of pulmonary hypoplasia and congenital diaphragmatic hernia. *Micr Res Techn* 1993; **26**: 389–399.
25. Zijlstra FJ, Vincent JE, Mol WM, Hoogsteden HC, Van Hal PThW. Eicosanoid levels in bronchoalveolar lavage fluid of young female smokers and non-smokers. *Eur J Clin Invest* 1992; **22**: 301–306.
26. Agostini E, Taglietti A, Agostini F, Setnikar I. Mechanical aspects of the first breath. *J Appl Physiol* 1958; **13**: 344–348.
27. Tenbrinck R, Scheffers EC, IJsselstijn H, Tibboel D, Lachmann B. Nitrofen induced diaphragmatic hernia: pressure-volume registration and artificial ventilation in newborn rats. *Applied Cardiopulmonary Pathophysiology* 1995; **5**: 257–264.
28. Steinhorn RH, Millard SL, Morin III FC. Persistent pulmonary hypertension of the newborn. Role of nitric oxide and endothelin in pathophysiology and treatment. *Clin Perinatol* 1995; **22**: 405–427.
29. Bos AP, Tibboel D, Koot VCM, Hazebroek FWJ, Molenaar JC. Persistent pulmonary hypertension in high-risk congenital diaphragmatic hernia patients: incidence and vasodilator therapy. *J Pediatr Surg* 1993; **28**: 1463–1465.
30. Bindl L, Fahnenstich H, Peukert U. Aerosolised prostacyclin for pulmonary hypertension in neonates. *Arch Dis Child* 1994; **71**: F214–F216.
31. Weir EK, McMurthy IF, Tucker A, Reeves JT, Grover RF. Prostaglandin synthetase inhibitors do not decrease hypoxic pulmonary vasoconstriction. *J Appl Physiol* 1976; **41**: 714–718.
32. Abman SH, Shanley PF, Accurso FJ. Failure of postnatal adaptation of the pulmonary circulation after chronic intrauterine pulmonary hypertension in fetal lambs. *J Clin Invest* 1989; **83**: 1849–1858.
33. Cott GR, Westcott JY, Voelkel NF. Prostaglandin and leukotriene production by alveolar type II cells in vitro. *Am J Physiol* 1990; **258**: L179–L187.
34. Brandsma AE, Ten Have-Opbroek AAW, Vulto IM, Molenaar JC, Tibboel D. Alveolar epithelial composition and architecture of the late fetal pulmonary acinus: an immunohistochemical and morphometric study in a rat model of pulmonary hypoplasia and congenital diaphragmatic hernia. *Exp Lung Res* 1994; **20**: 491–515.

ACKNOWLEDGEMENTS. The authors want to thank M. van Aken (Center for Animal Research, Erasmus University Rotterdam) for excellent technical assistance. This work was financially supported by the Netherlands Asthma Foundation, project number 91.56.

**Received 23 October 1996; accepted 26 November 1996**
